# Detection of Androgen Receptor Variant 7 (*ARV7)* mRNA Levels in EpCAM-Enriched CTC Fractions for Monitoring Response to Androgen Targeting Therapies in Prostate Cancer

**DOI:** 10.3390/cells8091067

**Published:** 2019-09-11

**Authors:** Claudia Hille, Tobias M. Gorges, Sabine Riethdorf, Martine Mazel, Thomas Steuber, Gunhild Von Amsberg, Frank König, Sven Peine, Catherine Alix-Panabières, Klaus Pantel

**Affiliations:** 1 Department of Tumor Biology, University Medical Center Hamburg-Eppendorf, 20246 Hamburg, Germany; c.hille@uke.de (C.H.); t.gorges@uke.de (T.M.G.); s.riethdorf@uke.de (S.R.); 2 Laboratory of Rare Human Circulating Cells (LCCRH), University Medical Centre of Montpellier–UM EA2415, 34295 Montpellier, France; martine-mazel@chu-montpellier.fr (M.M.); c-panabieres@chu-montpellier.fr (C.A.-P.); 3 Martini Clinic, University Medical Center Hamburg-Eppendorf, 20246 Hamburg, Germany; steuber@uke.de; 4 Department of Hematology and Oncology, University Medical Center Hamburg-Eppendorf, 20246 Hamburg, Germany; g.von-amsberg@uke.de; 5 ATURO, Urology Practice, 14197 Berlin, Germany; frank.koenig@aturo.berlin; 6 Department of Transfusion Medicine, University Medical Center Hamburg-Eppendorf, 20246 Hamburg, Germany; s.peine@uke.de

**Keywords:** prostate cancer, biomarkers, circulating tumor cells, androgen receptor, ARV7, abiraterone, enzalutamide

## Abstract

Expression of the androgen receptor splice variant 7 (*ARV7)* in circulating tumor cells (CTCs) has been associated with resistance towards novel androgen receptor (AR)-targeting therapies. While a multitude of ARV7 detection approaches have been developed, the simultaneous enumeration of CTCs and assessment of *ARV7* status and the integration of validated technologies for CTC enrichment/detection into their workflow render interpretation of the results more difficult and/or require shipment to centralized labs. Here, we describe the establishment and technical validation of a novel *ARV7* detection method integrating the CellSearch^®^ technology, the only FDA-cleared CTC-enrichment method for metastatic prostate cancer available so far. A highly sensitive and specific qPCR-based assay was developed, allowing detection of *ARV7* and *keratin 19* transcripts from as low as a single *ARV7^+^/K19^+^* cell, even after 24 h of sample storage. Clinical feasibility was demonstrated on blood samples from 26 prostate cancer patients and assay sensitivity and specificity was corroborated. Our novel approach can now be included into prospective clinical trials aimed to assess the predictive values of CTC/ARV7 measurements in prostate cancer.

## 1. Introduction

Prostate cancer (PCa) remains the second most commonly diagnosed cancer among men worldwide with an estimated 1.3 million new cases each year [1]. In contrast to other cancer types such as pancreatic cancer, routine preventive medical screens for PCa are accessible to a broad spectrum of the public and have been widely accepted, leading to a drastic increase of newly diagnosed PCa cases. Tissue biopsies are invasive and can be associated with adverse effects for the patient [2]. Furthermore, routine tissue biopsy is challenging in metastatic PCa (mPCa). In recent years, minimally invasive liquid biopsies, focusing on the identification of circulating tumor cells (CTCs) and circulating nucleic acids (ctDNA, miRNA) from whole blood samples, have gained tremendous attention [3,4,5,6,7]. While the prognostic relevance of CTCs in PCa, especially in the metastatic setting, has been thoroughly shown in large clinical trials [8,9,10,11], predictive value of CTC analysis and their clinical utility are still being debated [12,13,14,15,16,17]. While a multitude of therapeutic approaches exist, aimed at treating PCa in various disease stages, a subset of patients develop aggressive PCa subtypes that defy current therapeutic options. Therefore, simple detection of PCa is not sufficient and robust biomarkers are urgently needed to discern aggressive subtypes from clinically well treatable cancers, preferably without exposing patients to unnecessary tissue biopsies.

With the advent of novel hormone therapies such as enzalutamide and abiraterone and the emergence of innate and acquired resistance towards these therapies, the androgen receptor splice variant 7 (ARV7) has become a leading target of CTC research in PCa [17,18,19]. Multiple studies indicate that *ARV7* mRNA and ARV7 protein expression in CTCs is associated with resistance towards novel hormone therapies [20,21,22,23,24,25] and that *ARV7* expressing patients benefit more from taxane-based therapy [25,26,27]. This implicates ARV7 as a possible treatment selection biomarker for PCa patients prior to receiving novel hormone therapy (e.g., enzalutamide, abiraterone). Additionally, the ARV7 status is subject to change during therapy regimens [25,28,29], underlining the benefit of sequential sampling which becomes possible through liquid biopsy. ARV7 could therefore also represent a biomarker to monitor treatment response and predict upcoming therapy resistance.

While many approaches have been developed to assess ARV7 either on protein or mRNA level [20,24,30], only very few of these approaches allow for parallel CTC enumeration and morphological characterization while giving information on ARV7 status for individual CTCs [24,31], a limitation recently highlighted [32]. Additionally, even fewer were designed to use the only FDA-cleared CTC enrichment and detection technology shown to have clinical prognostic relevance in prostate cancer, the CellSearch^®^ system [33]. Here, we aimed to develop a protocol for *ARV7* detection using the CellSearch^®^ technology. With our novel workflow we were able to detect *ARV7* mRNA in as low as one CTC in 7.5 mL of whole blood.

## 2. Materials and Methods

### 2.1. Cancer Cell Lines

The human prostate cancer cell lines 22Rv1 (ATCC^®^ CRL-2505), VCaP (ATCC^®^ CRL-2876), LNCaP (ATCC^®^ CRL-1740) and PC3 (ATCC^®^ CRL-1345) were obtained from the American Type Culture Collection (ATCC, Manassas, VA, USA) and cultured according to ATCC recommendations.

LNCaP and 22Rv1 cells were cultured in RPMI 1640 medium, while the VCaP and PC3 cells were maintained in Dulbecco’s Modified Eagle Medium (DMEM). Media were additionally fortified with 10% fetal calf serum (FCS) (Gibco—Life Technologies, Darmstadt, Germany), 1% L-glutamine (Gibco—Life Technologies, Darmstadt, Germany) and 1% penicillin/streptomycin (Gibco—Life Technologies, Darmstadt, Germany), as recommended by ATCC. Cells were cultured in 25 cm^2^ flasks at 37 °C in a humidified atmosphere containing 5% CO_2_.

### 2.2. Blood Collection and Processing

Male healthy donor (HD) and patient blood samples were acquired in accordance to the World Medical Association Declaration of Helsinki and the guidelines for experimentation with humans by the Chambers of Physicians of the State of Hamburg (“Hamburger Ärztekammer”). All patients gave informed, written consent prior to blood collection (Ethics Approval: PV3779). Samples were drawn from 26 metastatic prostate cancer (mPCa) patients into standard 7.5 mL ethylenediaminetetraacetic acid (EDTA) vacutainers or CellSave^®^ (Menarini-Silicon Biosystems, Florence, Italy) preservation tubes respectively. Each patient therefore provided a matched sample of EDTA-KE (Sarstedt, Rheinbach, Germany) and CellSave^®^ blood for further analysis. CTCs from EDTA blood samples were enriched via the CellSearch^®^ Profile Kit (Menarini-Silicon Biosystems, Florence, Italy) and further analyzed for *ARV7* expression as described below. Samples collected into CellSave^®^ blood preservation tubes were processed via the CellSearch^®^ CXC-Kit (FITC labelled pan-keratin) [34]. Phycoerythrin labelled androgen receptor CellTracks Anti-Androgen Receptor (Janssen Diagnostics) antibody (10 µg/mL) was used for full-length AR (AR-FL) detection in the fourth channel of the CellSearch^®^ for 12/26 mPCa patients. All analyses were performed by trained CellSearch^®^ analysist. CTCs were defined as keratin positive and CD45 negative cells with a nuclear DAPI staining.

### 2.3. Spiking of Healthy Donor Blood

For spiking experiments, cell line cells were washed once with 1 x PBS (Gibco-Life Technologies, Darmstadt, Germany) and treated with 0.25% trypsin-EDTA (Gibco-Life Technologies, Darmstadt, Germany) for 5 min at 37 °C prior to being resuspended in culture medium. The cell suspension was centrifuged at 190× *g* for 5 min after which the supernatant was discarded and the cells were again resuspended in fresh culture medium. The cells were spread to a petri dish filled with corresponding medium, manually counted and picked under a light microscope. Defined cell counts were added directly to healthy donor blood samples.

### 2.4. Immunocytochemical Stainings on Cell Culture Plates

Cells were seeded into 24-well plates at the rate of 50,000 cells/well, and maintained at 37 °C in a humidified atmosphere containing 5% CO_2_ until reaching 80% confluence. Cells were then fixed and permeabilized using IntraPrep Permeabilization Reagent (A07803, Beckman Coulter, Brea, CA, USA), and blocked with 10% Goat serum for 1 h at room temperature. Cells were subsequently incubated with (i) primary antibodies Anti-AR (AR-V7 specific) antibody [EPR15656] (Abcam, Cambridge, United Kingdom) at a final concentration of 10 µg/mL, or (ii) Rabbit IgG, monoclonal [EPR25A]-Isotype Control (Abcam, Cambridge, United Kingdom) (our negative control) at a final concentration of 10 µg/mL. All wells were also incubated with the anti-PanCK^PE^ (Menarini-Silicon Biosystems, Florence, Italy). Following this first incubation, cells were washed with 1% goat serum in PBS, incubated with the FITC-conjugated secondary antibody (1:20 in PBS containing 10% Goat serum), and washed twice with 1% goat serum in PBS.

In parallel, the presence of the androgen receptor (AR) was tested using the anti-AR^AF488^ [D6F11] XP Rabbit antibody (0.5 µg/mL, Ozyme, Saint Cyr L’Ecole, France); in the control wells, the Rabbit [DA1E] IgG^AF488^ XP isotype (0.5 µg/mL, Ozyme, Saint Cyr L’Ecole, France) was used. Cell imaging was obtained under 20x magnification using a Fluorescent Axio Observer microscope (Carl Zeiss, Oberkochen, Germany).

### 2.5. Immunocytochemical Stainings on Cytospins

Cell suspensions of selected prostate cancer cell lines (22Rv1, LNCap, PC3) were spun down on glass slides (190× *g*, 5 min) and dried at room temperature (RT) over night. Cells were subsequently fixed and permeabilized using the respective CellSearch CXC Kit reagents (Menarini-Silicon Biosystems, Florence, Italy) and blocked with 10% AB-Serum (BioRad, Rüdigheim, Germany). Primary antibodies targeting ARV7, 4 µg/mL of clone AG10008 (unlabeled, Precision, Columbia, Maryland, USA) and 6 µg/mL EPR15656 (unlabeled, Abcam, Cambridge, United Kingdom) were tested. Secondary antibodies were applied and contained a DAPI nuclear counterstain. Secondary rabbit-anti mouse (Alexa 546, polyclonal, Thermo Fisher Scientific, Dreieich, Germany) and mouse-anti-rabbit (Alexa 546, polyclonal, Thermo Fisher Scientific, Dreieich, Germany) antibodies were used. Cytospins were covered in Prolong Gold Antifade Reagent (Thermo Fisher Scientific, Dreieich, Germany) and cover slipped for analysis. Slides were manually assessed using a fluorescence microscope (Axioplan 2, Carl Zeiss, Oberkochen, Germany).

### 2.6. Western Blots

Cell lines (22Rv1, VCaP, LNCaP, and PC3) were cultured to 70% confluency, harvested in urea lysis buffer (9.8 M Urea, 15 mM EDTA, 30 mM Tris) and homogenized by ultrasonic treatment. Protein concentration was measured with the Pierce BCA Protein Assay Kit (Pierce, Rockford, Illinois, USA). 40 µg of total protein was applied for Western Blot analysis for each respective cell line alongside pre-stained peqGold protein marker-V (VWR, Erlangen, Germany). Proteins were separated according to size using a Laemmli buffer system and 8% polyacrylamide separation gel. Two ARV7 antibodies, mouse-AG10008 (Precision, Columbia, MD, USA; 2 µg/mL) and rabbit-EPR15656 (Abcam, Cambridge, United Kingdom; 1.5 µg/mL) were applied in dilutions according to the supplier’s instruction manual in 5% milk powder. Alpha-tubulin was used as a loading control (Cell Signaling Technology, Danvers, MA, USA). Species specific secondary antibodies (horseradish peroxidase conjugated, DAKO, Glostrup, Germany) were applied at 1:10.000 dilution in 5% milk powder. Protein bands were visualized using SignalFire™Plus ECL reagent (Cell Signaling Technology, Danvers, MA, USA) and X-ray films (CEA, Hamburg, Germany) according to the instruction manual.

### 2.7. RNA Extraction and cDNA Synthesis

For cell line characterization and PCR establishment RNA was isolated from prostate cancer cell lines grown in a T25 culture flask at 70% confluency using the NucleoSpin^®^ RNA isolation kit (Macherey-Nagel, Düren, Germany) according to manufacturer’s instructions. RNA concentration and purity were controlled via NanoDrop 1000 spectrophotometer (Thermo Fisher Scientific, Dreieich, Germany) following isolation. 0.5 µg of RNA per cell line were used for DNA synthesis with the First Strand cDNA Synthesis Kit (Thermo Fisher Scientific, Dreieich, Germany) according to manufacturer’s instructions. cDNA Synthesis was carried out in a PeqSTAR 96 Universal Gradient thermocycler (VWR International, Darmstadt, Germany).

Following CTC enrichment via the CellSearch^®^ Profile Kit (Menarini-Silicon Biosystems, Florence, Italy) samples were transferred to a fresh 1.5 mL tube (Sarstedt, Rheinbach, Germany). To do so, a 1000 µL pipette tip was first coated with a solution of 0.1 mg/mL of BSA/PBS to circumvent binding and sticking of CTCs to the pipette surface. All RNA work was performed using sterile, DNA/RNA-free, filtered Biosphere^®^ plus pipette tipps (Sarstedt, Rheinbach, Germany). The Profile^®^ sample tube was washed with 500 µL of 1x DPBS (cell culture use) (Thermo Fisher Scientific, Dreieich, Germany), which was also added to the sample. Subsequently the sample was placed in a magnetic rack (Magnetcellect; R&D systems, Minneapolis, MN, USA) for 10 min. The supernatant was discarded, and the sample was washed with 1000 µL of 1x DPBS, followed by another 10 min attached to the magnetic rack. This step was repeated with 500 µL of 1x DPBS prior to resuspension of the Profile^®^ beads in 150µL of lysis buffer (Dynabeads mRNA DIRECT Kit; Thermo Fisher Scientific, Dreieich, Germany). Samples were immediately frozen at −80 °C. Sample lysates were stored for a maximum of 14 days prior to RNA isolation and cDNA synthesis.

For RNA extraction, the Dynabeads mRNA DIRECT Kit (Thermo Fisher Scientific, Dreieich, Germany) was applied according to manufacturer’s instructions. Following the last wash step with Wash buffer B, supernatant was removed, and beads were resuspended in 14.75 µL of Nuclease-free H_2_O (Qiagen, Hilden, Germany) and placed in a PCR cycler at 75 °C for 5 min to ensure elution of mRNA from the beads. Subsequently cDNA was synthesized using the Sensiscript Reverse Transcription Kit (Qiagen, Hilden, Germany) with Recombinant Rnasin^®^ (Promega, Mannheim, Germany) as an added RNase inhibitor. Primer addition was not necessary as the contained dynabeads function as oligo-dT primers. RNase inhibitor was limited to 0.25 µL, leading to a total mastermix of 5.25 µL added to each RNA sample (total reaction volume of 20 µL). Following cDNA synthesis, beads were removed via magnet and supernatant was transferred to a fresh PCR tube for subsequent qPCR analysis.

### 2.8. Polymerase-Chain Reaction (PCR) Analysis

For *AR-FL* and *ARV7* primer evaluation, 10 ng of cDNA of each prostate cancer cell line was applied per PCR. The PCR reaction conditions for initial primer testing were adapted from the original Antonarakis et al. publication by the Johns Hopkins Group [20]. Reactions were run in a PeqSTAR 96 Universal Gradient thermocycler (VWR International, Darmstadt, Germany).

PCR primer pairs (Sigma Aldrich, Steinheim, Germany) chosen for PCR targeted *AR-FL* (fw-CAGCCTATTGCGAGAGAGCTG, rev-GAAAGGATCTTGGGCACTTGC, fragment size of 125 bp) [20] and *ARV7* (Antonarakis et al. [20]: fw-CCATCTTGTCGTCTTCGGAAATGTTA, rev-TTTGAATGAGGCAAGTCAGCCTTTCT, fragment size of 125 bp; Guo et al. [35]: fw-CTACTCCGGACCTTACGGGGACATGCG, rev-TGCCAACCCGGAATTTTTCTCCC, fragment size of 314 bp; Liu et al. [36]: fw- CAGGGATGACTCTGGGAGAA, rev- GCCCTCTAGAGCCCTCATTT, fragment size of 112 bp; UKE: fw-AGAAAGGCTGACTTGCCTCA, rev- CGCCAGGTTTCTCCAGACTA, fragment size of 73 bp) gene sequences. Novel UKE primers were designed using the Primer 3 software [37]. Primers were aliquoted at stock concentrations of 100 µM with Nuclease-free H_2_O (Qiagen, Hilden, Germany) and frozen at −20 °C. Final concentrations of 10 µM were applied to PCRs.

To visualize PCR products, they were mixed with DNA Gel loading dye (6x) (Thermo Fisher Scientific, Dreieich, Germany) and applied to 2% agarose gels containing GelRed^®^ Nulceic Acid Gel Stain (Biotum, Fremont, CA, USA) at 1/µL per ml of agarose gel. The Quick-Load^®^ 100 bp DNA Ladder (New England Biolabs, Frankfurt am Main, Germany) was used as a size standard. PCR fragments were visualized using the Gene Genius bioimaging system (Syngene, Bangalore, India).

### 2.9. Quantitative Polymerase-Chain Reaction (qPCR) Analysis

qPCRs were pipetted under a separate flow hood with sterile, DNA/RNA-free, filtered Biosphere^®^ plus pipette tipps (Sarstedt, Rheinbach, Germany) and performed in a CFX96 Touch™ Real Time PCR Detection System (BioRad, Rüdigheim, Germany). Maxima SYBR-Green fluorescent dye (Thermo Fisher Scientific, Dreieich, Germany) was used for product detection. Amplification was performed under the following conditions: after an initial denaturation step (10  min at 95 °C), 40 amplification cycles were carried out, consisting of denaturation at 95 °C for 30 seconds, annealing at 60 °C for 30  s, and elongation for 30 s at 72 °C. A final elongation step at 72 °C (10  min) was followed by a melting curve analysis and storage of the samples at 4 °C. Data was summarized and converted into Excel files using the CFX Manager Software (BioRad, Rüdigheim, Germany). For qPCR analysis, two additional primer sets targeting *K19* (fw-CGAACCAAGTTTGAGACGGA; rev-GATCTGCATCTCCAGGTCGG; fragment size of 117 bp) and *Actin* (x) gene sequences were applied. Samples were applied in triplicates and average Cq values as well as standard deviations were calculated. Primers were aliquoted at stock concentrations of 100 µM with Nuclease-free H_2_O (Qiagen, Hilden, Germany) and frozen at −20 °C. Final concentrations of 10 µM were applied to qPCRs.

Relative gene expression of *AR-FL* and *ARV7* in initial primer testing and cell line characterization was normalized from data sets by the comparative Cq method [38]. Briefly, the first amplification cycle showing significant increase of fluorescence signal over background level was defined as the cycle of quantification (Cq). Cq data of *AR-FL* and *ARV7* was normalized by subtracting the Cq value of *Actin* from the respective target gene for each cell line tested, generating a ΔCq value. Subsequently, the ΔΔCq values were calculated by subtracting the ΔCq of each specific gene calculated for the different cell lines (22Rv1, VCap, and LNCaP) from the ΔCq values calculated for gene expression in PC3 cells. Finally, ΔΔCq values were converted to log2 fold changes by applying 2^- ΔΔCq^_._ Ten nanograms of cDNA were applied per triplicate well.

Following RNA isolation and cDNA transcription from CellSearch^®^ Profile Kit (Menarini-Silicon Biosystems, Florence, Italy) enriched samples, the 20 µL of cDNA mix was applied in triplicates for each gene (2 µL/well). No absolute quantification or normalization of genes was performed as levels of *Actin* gene expression is variable depending on background leucocyte cDNA co-amplified following CTC enrichment. Gene expression was confirmed when at least 2/3 triplicates showed detectable transcript levels under a Cq threshold of 35. Quality of the results was furthermore corroborated by melting curve analysis and subsequent visualization of amplified products on 2% agarose gels (see above).

## 3. Results

### 3.1. Test of Commercially Available ARV7 Antibodies for Fourth CellSearch^®^ Channel

To allow assessment of ARV7 protein levels in parallel to CTC enumeration on a cell-specific level, we initially tested available ARV7 antibodies with the aim of adding them to the fourth channel of the CellSearch^®^ system. Currently only few commercial antibodies are available, aimed at detecting ARV7 protein either via immunohistochemistry (IHC), immunocytochemistry (ICC) and/or western blot.

**Figure 1:**
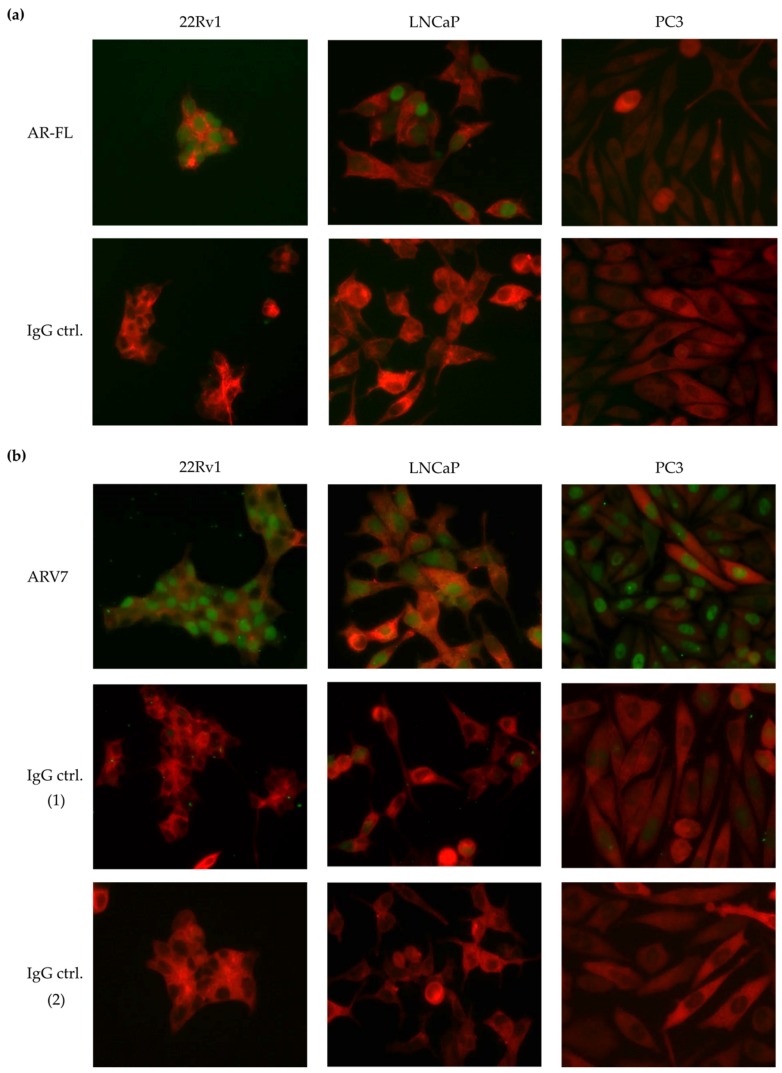
Immunocytochemical (ICC) staining of full-length androgen receptor (AR-FL) and ARV7 on three selected prostate cancer cell lines. Cells are stained for pan-keratin in red (anti-PanCK, CellSearch^®^, Menarini) in all images. (**a**) Upper panel: ICC staining performed using the anti-AR-FL antibody (7395S Ozyme) in green. AR-FL positive cells lines 22Rv1 and LNCaP show positive nuclear AR-FL staining, while PC3 cells remain unstained. Lower panel: ICC control staining using Rabbit [DA1E] IgG XP isotype (2975S Ozyme) in green showing the absence of unspecific staining on 22RV1, LNCaP and PC3 cells. (**b**) Upper panel: ICC staining performed using the anti-ARV7 antibody [EPR15656] (209491 Abcam) detected by a FITC-conjugated secondary antibody. A positive nuclear staining is observable on all three cell lines (in green), indicating unspecific signal of the antibody in PC3. Medium panel: ICC staining performed with the Rabbit IgG, monoclonal [EPR25A]-Isotype Control (172730 Abcam) detected with FITC-conjugated secondary antibody (97050, Abcam) showing negativity on 22RV1, LNCaP, and PC3 cells. Lower panel: ICC staining performed with FITC-conjugated secondary antibody (97050, Abcam) showing negativity on 22RV1, LNCAP, and PC3 cells.

Three established prostate cancer cell lines were chosen for method establishment, each cell line representing a specific status of AR-full length (AR-FL) and ARV7 protein expression: 22Rv1 (AR-FL^+^/AR-V7^+^), LNCaP (AR-FL^+^/AR-V7^+/−^), and PC3 (AR-FL^−^/AR-V7^−^). First, the cell lines were characterized for AR-FL (Figure 1a), resulting in cell line specific nuclear ICC staining (22Rv1 and LNCaP) or absence of staining (PC3) for the full-length protein, seen in green. Next, we tested the anti-ARV7 antibody [EPR15656] described in literature to specifically stain nuclear ARV7 [25]. This antibody did not result in cell line specific staining results, as all three tested cell lines including the ARV7^-^ PC3 cells showed green nuclear ARV7 staining (Figure 1b). Similar results were obtained using the antibody [EPR15656] as well as an additional commercially available ARV7 antibody on cell line cytospins (Figure S1). In western blot analysis the anti-ARV7 antibody [AG10008] by Precision showed cell line specific results, correctly detecting 22Rv1 and VCaP lysate as ARV7^+^, LNCaP protein levels as below detection limit and identifying PC3 cells as ARV7^-^ (Figure S1a). In contrast, the anti-ARV7 antibody [EPR15656], showed an unspecific western blot signal for PC3 cells (Figure S1a). In ICC both antibodies failed to correctly characterize the chosen prostate cancer cell lines, giving unspecific staining results (Figure S1b,c).

In conclusion, none of the tested antibodies were deemed suitable for characterization of ARV7 protein on CTCs via the CellSearch^®^ system. Additionally, the most intensively tested anti-ARV7 antibody [EPR15656] [25], described to give a specific nuclear and unspecific cytoplasmic staining did not show reliable results in our hands (Figure 1b), giving unspecific nuclear staining signals in ARV7^−^ PC3 cells, even when neglecting the cytoplasmic staining and considering the described, relevant nuclear staining.

### 3.2. Development of a qPCR Based Assay to Detect ARV7 mRNA

**Figure 2:**
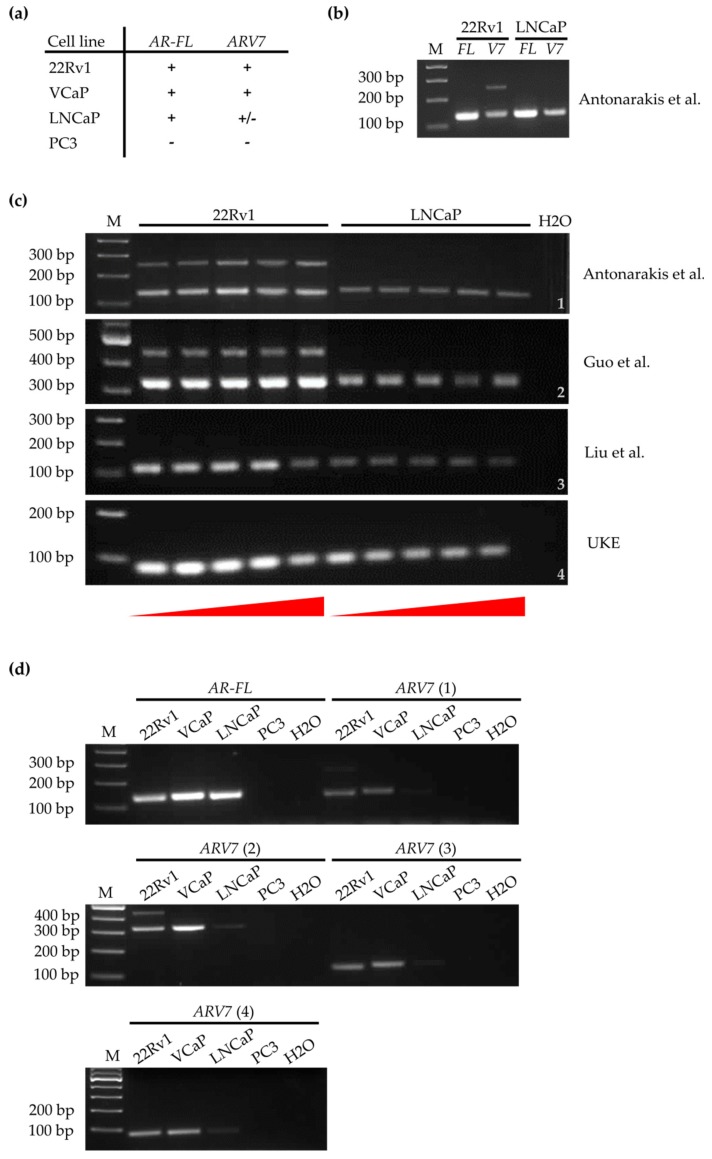
PCR-based detection of *AR-FL* and androgen receptor splice variant 7 (*ARV7*) in selected prostate cancer cell lines. Letter M indicating DNA ladder (marker) lanes. Ten nanograms of cDNA were analyzed for each PCR sample. (**a**) Schematic overview of *AR-FL* and *ARV7* positivity (+) and negativity (−) for established prostate cancer cell lines, as described in literature. (**b**) Agarose gels of a PCR detecting *AR-FL* and *ARV7* in cDNA isolated from 22RV1 and LNCaP cells. *ARV7* cDNA was detected using the primers described by Antonarakis et al. [20]. PCRs were performed for 39 cycles. 125 bp PCR products are expected for both *AR-FL* and *ARV7*. The *ARV7* PCR shows an additional, uncharacterized band at between 250–300 bp for 22RV1 cells, but not for LNCaP cells. (**c**) Agarose gel of a gradient PCR for *ARV7* on 22RV1 and LNCaP cDNA using different primer pairs. PCRs were performed for 39 cycles. Temperatures increasing from 58.5 °C to 65.5 °C, indicated by red triangles below gel images. Antonarakis (1) and Guo (2) primers both show secondary PCR bands on 22Rv1 cDNA (between 200–300bp and between 400–500 bp, respectively). Liu (3) and UKE (4) primers both give expected PCR bands for *ARV7* at 112 bp and 73 bp. Signal intensity appears higher, possibly indicating more generated PCR product, for UKE primers. (**d**) Agarose gels of PCRs detecting *AR-FL* and *ARV7* in cDNA of 22RV1, VCaP, LNCaP, and PC3 prostate cancer cell line cells. PCRs were performed for 30 cycles. AR-FL primer set, results in specific PCR signals in AR^+^ and AR^–^ cell lines. *ARV7 (1)* corresponds to Antonarakis et al., *ARV7 (2)* corresponds to Guo et al., *ARV7 (3)* corresponds to Liu et al., and *ARV7 (4)* corresponds to our newly developed UKE primer sets.

As an alternative to protein-based detection we subsequently aimed at establishing a qPCR-based approach to detect *ARV7* on an mRNA level. We added an additional prostate cancer cell line to the analysis, to further confirm the robustness of our method. VCaP cells show similar *AR-FL* and *ARV7* expression profiles as 22RV1 cells (*AR-FL*^+^/*AR-V7*^+^) and were used as a second *ARV7*^+^ cell line during method establishment. An overview of the *AR-FL* and *ARV7* status for all four cell lines is listed in Figure 2a. Initially, we planned on using the *AR-FL* and *ARV7* primer sets already published [20] for our qPCR-based detection and then modifying the CTC pre-enrichment steps. However, when testing the primers using PCR according to the published protocol, it became clear that while the *AR-FL* primers showed specific bands at the correct expected size of 125 bp (Figure 2b), an additional, undescribed PCR fragment of around 250 bp was detected using the *ARV7* primers in 22Rv1 but not in LNCaP cells (Figure 2b). To ensure optimal primer quality for *ARV7* detection, additional *ARV7* primer sets described in literature [35,36] as well as an own design (UKE), were employed. To exclude that the unspecific PCR fragments detected were generated due to incorrect annealing temperature or incorrect cDNA synthesis, we tested all four primer sets in a gradient PCR on freshly generated 22Rv1 and LNCaP cDNA (Figure 2c). Again, an additional PCR product was detected for the Antonarakis [20] and Guo [35] primer sets across all annealing temperatures in 22Rv1 cells but not LNCaP cells (Figure 2c, lines 1,2). This could represent an additional AR splice variant, similar to *ARV7* [30]. Using the original protocol of 40 amplification cycles [20,23] this additional transcript could come up in clinical samples, especially those with high CTC counts, and result in an unaccounted bias. In contrast, the Liu [36] and UKE primer sets, resulted in specific PCR fragments at 112 bp and 73 bp, respectively (Figure 2c, lines 3,4). The fragment signal intensity appeared slightly higher for the UKE primers (Figure 2c, line 4) in comparison to the Liu primers [36] (Figure 2c, line 3), which could indicate a higher amount of generated PCR product. However, this cannot be conclusively deduced from qualitative PCR. Decreasing the PCR cycles from 39 to 30 (Figure 2d), reduced the unspecific PCR signals down to hardly visible levels for the Antonarakis [20] and Guo [35] primer sets (Figure 2d). However, as quantitative PCR represents a much more sensitive method than qualitative PCR, both primer sets were discarded for further experiments. Both the Liu [36] and UKE primer sets displayed cell line specific PCR results and PCR fragments at correct sizes, resulting in further evaluation of these two primer sets via qPCR.

**Figure 3:**
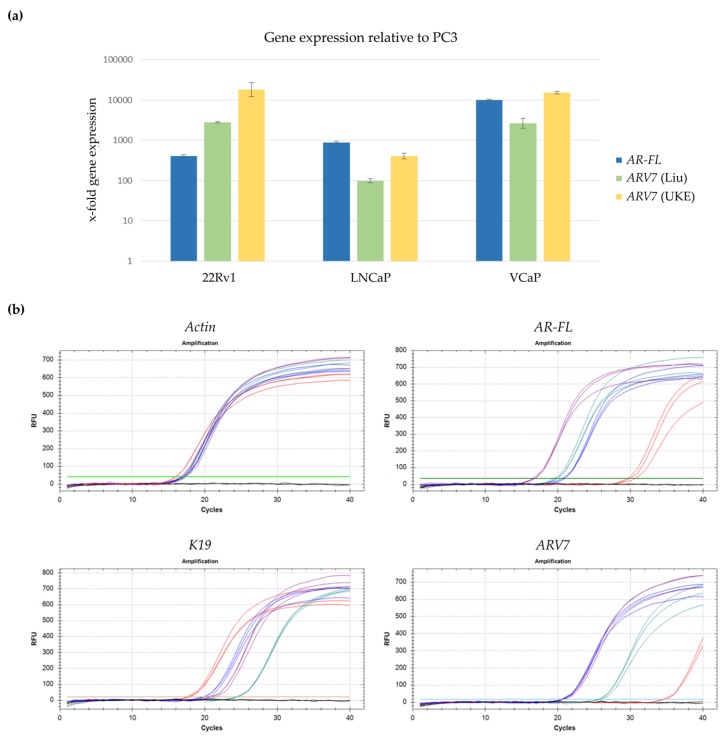
qPCR-based characterization of selected prostate cancer cell line cDNA. cDNA was generated from RNA isolated from 22RV1, VCaP, LNCaP, and PC3 cells and analyzed via qPCR. (**a**) Relative gene expression of *AR-FL* and *ARV7* using primers by Liu et al. and our newly developed primers (UKE). Gene expression was first normalized to actin and subsequently displayed relative to PC3 gene expression. Standard deviation is indicated as brackets. (**b**) Representative qPCR expression profiles for different target genes (*Actin, AR-FL, K19* and *ARV7*) across all four chosen cell lines: 22RV1 (blue), VCaP (purple), LNCaP (green), and PC3 (red). All samples were applied in triplicates.

Gene expression levels of *AR-FL* and *ARV7* (using the Liu and UKE primers) were assessed for 22Rv1, LNCaP, and VCaP cells in relation to their respective expression in PC3 cells (Figure 3a). As expected, both *AR-FL* and *ARV7* gene expression were dramatically increased in all three cell lines compared to PC3 cells. Additionally, the UKE primers showed most effective detection of *ARV7* (Figure 3a). All further experiments were therefore carried out using the newly designed UKE primers.

Apart from *AR-FL* and *ARV7, K19*, and *Actin* gene expression were also measured via qPCR. *Actin* functioning as a gene for normalization and a confirmation of successful cDNA synthesis, and *K19* as an established marker for CTC detection in blood [39,40] thus allowing confirmation of the presence of CTCs in future clinical samples. Figure 3b shows representative qPCR curves for all four cell lines (in different colors) for each gene. Due to the high sensitivity of qPCR analysis, *ARV7* expression can be detected at around 36 cycles for cDNA inputs generated from high PC3 cell counts (Figure 3b). This is an enormous difference to the approximately 22 cycles necessary for detection of ARV7^+^ cell lines (Figure 3b). Despite the fact that such high CTC cell counts are extremely rarely to be expected in clinical samples, a cut-off of ≤35 cycles was established for gene expression to be counted as positive for the analyzed genes in all further analysis.

### 3.3. Combining Profile-Kit-Based CTC Enrichment with ARV7 mRNA Detection

**Figure 4:**
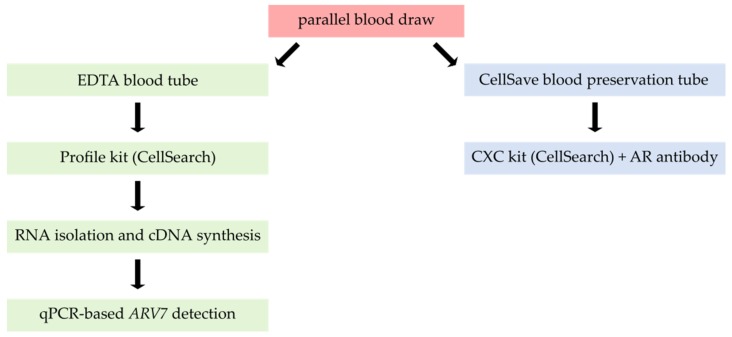
Schematic workflow of *ARV7* detection combined with parallel circulating tumor cell (CTC) enumeration.

To allow for use of the CellSearch^®^ system to isolate prostate cancer CTCs for *ARV7* detection on the one hand and enable parallel CTC quantification on the other, a two-armed approach was designed (Figure 4). 7.5 mL of whole blood was taken in parallel into standard EDTA tubes for RNA isolation and CellSave^®^ blood preservation tubes for CTC enumeration, respectively. From EDTA blood, CTCs were enriched via the CellSearch^®^ Profile Kit for subsequent RNA analysis. RNA was isolated and cDNA synthesized prior to analysis of *ARV7, K19,* and *Actin* via qPCR. In parallel CellSave^®^ preserved blood was processed using the CellSearch^®^ CXC Kit thus allowing for parallel AR-FL protein characterization in the fourth fluorescent channel of the device (Figure 4).

“Mock” samples were generated to mimic clinical sample handling. Differing amounts of *ARV7*^−^ and *ARV7*^+^ cell line cells were manually spiked into healthy donor (HD) blood and directly processed by our workflow (Table 1). Following the qPCR run, generated products were applied to a gel electrophoresis allowing final confirmation of gene expression status (data not shown).

All HD samples measured (n = 3) were *ARV7* and *K19* negative (Table 1). PC3 samples were negative for *ARV7* and positive for *K19*, confirming the specificity of the established assay. *ARV7* and *keratin 19* were still detectable down to 5 *ARV7*^+^ 22RV1 cells using our protocol (n = 3), demonstrating high sensitivity (Table 1).

### 3.4. Assessment of Sample Storage Parameters

mRNA instability represents a common issue for RNA analysis. Sample processing time frames and optimal blood collection tubes therefore need to be carefully assessed to allow for reliable mRNA detection. As cells are not fixed in EDTA blood tubes, which is essential for subsequent RNA isolation, potential CTCs could deteriorate over time. This is especially crucial when calculating time frames for shipment of clinical samples. EDTA blood spiked with cell lines was left at room temperature (RT) for 24 h (Table 2) and 48 h (Table 3), respectively, to test processing windows. Following the qPCR run, generated products were applied to a gel electrophoresis allowing final confirmation of gene expression status (data not shown).

After 24 h of sample storage at RT, 5 *ARV7*^+^ cells were still reliably detected using the assay (Table 2). This was confirmed on two ARV7^+^ cell lines (22Rv1 and VCaP). Additionally, as low as 3 and down to 1 *ARV7*^+^ cells were detectable (Table 2). With these low cell counts, detection frequency is more variable as cell enrichment from whole blood and extremely careful sample handling play crucial roles. Still, correct detection down to a single ARV7^+^ cell is possible. After 48 h, detection of *ARV7* and *K19* transcripts is subject to even higher fluctuation and increased cell counts would be needed to robustly detect transcripts of interest from these samples (Table 3). The specificity of our assay was demonstrated as no signals for *ARV7* or *K19* were seen in blood samples from healthy individuals in EDTA blood tested for 24h (3/3) as well as 48 h (3/3) of sample storage (Table 2 and Table 3).

Blood tube types vary and some may be more suitable for our assay than others. Therefore, we additionally tested the performance of AdnaCollect blood collection tubes (Qiagen, Hilden, Germany), designed for mRNA characterization by the AdnaTest Prostate Cancer (Qiagen, Hilden, Germany) with our assay. This tube has been used for PCR-based detection of RNA transcripts from whole blood and could therefore provide an alternative to EDTA, potentially prolonging the sample processing window. Again, different cell counts were spiked into HD blood, this time in AdnaCollect blood collection tubes, and processed after 48 h of storage with our protocol. In our hands, these tubes were able to detect *ARV7* in spiked samples, down to 5 ARV7^+^ cells (Table 3). However, as *ARV7* and *K19* signals were seen in all three tested HD samples (Table 3) indicating low specificity, the use of this blood tube type was not further continued.

Our protocol ensures specific detection of tumor cell transcripts in 7.5 mL of blood down to a single cell level even after 24 h of sample storage (Table 2). Conclusively, a sample preparation window of 24 h was determined for the evaluation of clinical samples taken into EDTA blood to allow for sample shipment while ensuring robust detection of *ARV7* from CTCs.

### 3.5. Clinical Feasibility of the Complete ARV7 Detection Workflow

The clinical feasibility of our assay was demonstrated by analyzing blood samples of 26 metastatic prostate cancer (mPCa) patients. Detailed clinical patient data is listed in Table S1. qPCR based *ARV7* analysis was performed within 24 h of sample collection from 7.5 ml of EDTA blood for all 26 patients. Parallel blood draws to assess CTC counts via CellSearch^®^ were collected and processed for 23/26 patients. AR-FL staining in the fourth fluorescent channel was available for 12/23 patient samples processed via CellSearch^®^ (Table 4).

**Figure 5:**
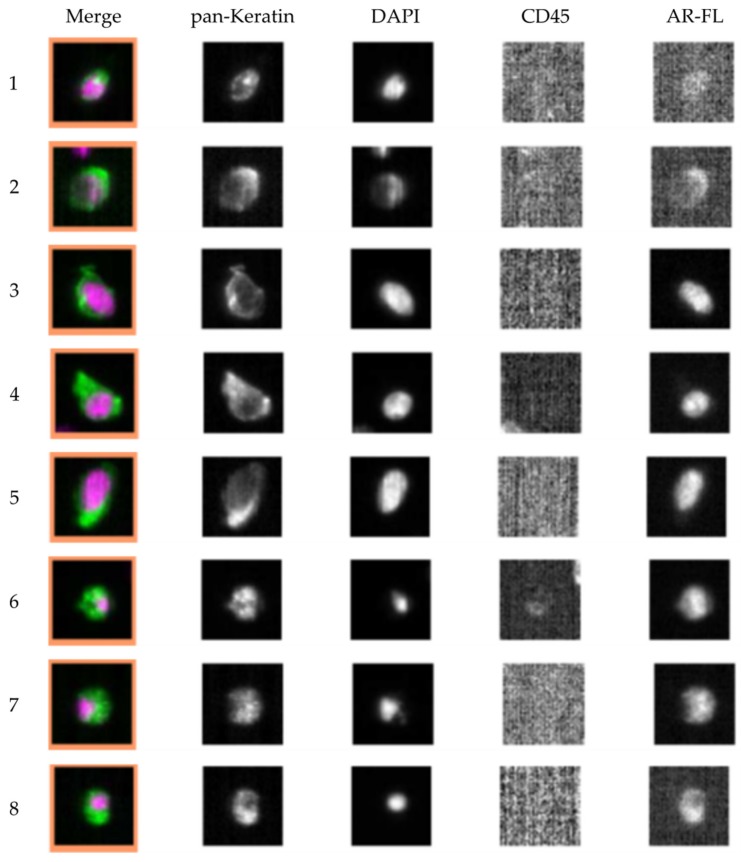
Representative CellSearch^®^ images of CTCs and AR-FL staining from a single prostate cancer patient case. This patient had detectable AR-FL negative (**1**), weakly positive (**2**), nuclear AR-FL positive (**3**–**5**) and cytoplasmatically AR-FL positive (**6**–**8**) CTCs.

Of the patient samples analyzed via CellSearch^®^ 86.2% (19/23) were found to have ≥1 CTC in 7.5 mL of blood. In 52.2% (12/23) of patients ≥5 CTCs were detected in 7.5 ml of whole blood, reaching the clinically prognostic cut-off value for worse overall survival for metastatic mPCa patients [8]. The median of detected CTCs for our cohort is 6 (range: 0–398 CTCs) and the average is 32 CTCs/7.5 mL of blood. *ARV7* mRNA was detected in 46.2% (12/26) of mPCa patients, *K19* was detected is 57.7% (15/26) of samples and *Actin* was detected in all samples (26/26), indicating effective cDNA transcription. Four measured patients were negative for the androgen receptor splice variant and positive for *K19* (e.g., samples UKE-10 and UKE-11). Additionally, one patient was positive for *ARV7* expression and negative for *K19* (UKE-23). No *ARV7* or *K19* gene expression was found in samples classified as CTC negative by the CellSearch^®^ system. Evaluation of the first 26 clinical samples resulted in 42.3% of *ARV7^+^*/*K19*^+^ of all cases (11/26) and 52.6% of *ARV7^+^*/*K19*^+^ cases (10/19) with ≥1 detectable CTC. Representative CellSearch^®^ images of AR-FL staining are shown in Figure 5.

Only three of the 12 cases in which AR-FL protein staining was assessed in the CellSearch^®^ (UKE-9, UKE-14, UKE-17) had detectable nuclear AR-FL protein levels (Table 4). Two of these three patients (UKE-14, UKE-17) had a mixed CTC population of nuclear and cytoplasmic AR-FL^+^ CTCs. Of these three patients, two were *ARV7* negative with our assay (UKE-9, UKE-14). Additional five patients showed cytoplasmic AR-FL protein expression, more than half of these patients were ARV7 positive (3/5).

For the majority of patient samples tested (88.4% or 23/26), the CTC count as measured by the CellSearch^®^ system was in accordance to *K19* detection in parallel samples (Table 4). Detection of *ARV7* was possible in 2/6 patients with only a single CTC detected in the patient’s blood (UKE-12, UKE-13) confirming the assays sensitivity (Table 4). *K19* was detected in 4/6 patients with only a single CTC indicating careful and effective sample handling (Table 4).

## 4. Discussion

The CellSearch^®^ Profile technology allows a reliable, standardized, and automated enrichment of EpCAM-positive cancer cells. ARV7 expression in CTCs of prostate cancer patients has been linked to resistance toward AR-targeted therapy, in particular enzalutamide and abiraterone [20,25]. Our novel approach ensures specific detection of *ARV7* transcripts in CTCs isolated by the CellSearch^®^ system down to the single cell level. The specificity of our assay was indicated as no signals for *ARV7* or *K19* were seen in 9 blood samples from healthy, male individuals (Table 1, Table 2 and Table 3). Our protocol ensures specific detection of tumor cell transcripts in 7.5 mL of blood even after 24 h of sample storage (Table 2). Robust *ARV7* and *K19* detection is feasible in as low as 5 *ARV7^+^/K19^+^* cells (Table 2). Transcript expression below 5 cells, even down to 1 *ARV7^+^/K19^+^* cell, was possible (Table 2). The clinical feasibility of our assay and its high sensitivity (down to a single CTC) was demonstrated in a cohort of 26 mPCa patients (Table 4).

Antonarakis et al. linked *ARV7* mRNA expression on CTCs of mCRPC patients receiving enzalutamide and/or abiraterone therapy to lower PSA response rates, as well as shorter progression free and overall survival [20]. Following this initial study, conducted with a combination of bead-based CTC enrichment and subsequent qPCR multiplexing, the group confirmed their finding in a larger cohort of 202 CRPC patients [23]. In their study, CTC^−^ patients were found to have the best outcome (judged by best PSA-response, PSA progression-free survival, progression-free survival, and overall survival), followed by CTC^+^/*ARV7*^−^ and finally CTC^+^/*ARV7*^+^ patients [23]. Additionally, it was demonstrated that *ARV7* status can change in the course of hormone therapy [25,28,29] and that within one patient *ARV7* status on CTCs can be heterogeneous [41].

The CellSearch^®^ system enables validated and automated enrichment of EpCAM-positive cancer cells [8,42,43,44,45]. Ideally, adding a specific and sensitive anti-ARV7 antibody to the fourth fluorescent channel of the CellSearch^®^ device would therefore represent a valuable alternative to allow parallel CTC enumeration and the assessment of ARV7 status for each respective CTC. Unfortunately, detection of ARV7 protein using the CellSearch^®^ technology was dramatically hampered by lacking specificity of most existing ARV7 antibodies (Figure 1, Figure S1). Recently, a novel commercially available antibody has been tested and validated for immunohistochemistry on primary tumor tissue, showing specific ARV7 staining results [32]. Whether this antibody might represent a promising novel candidate for immunocytochemical analysis and combination with CellSearch^®^ needs to be investigated in future studies. However, so far most sources of CTC-related ARV7 information stems from RNA measurements.

The meaningful clinical impact of *ARV7* expression of CTCs [20,46] has led to the development of a multitude of different assays targeting ARV7 protein [25] or *ARV7* transcripts [30,31,47,48]. Primarily the developed methods are based on the analysis of pooled lysate of an enriched CTC fraction [26,30,48], only few perform whole blood gene expression analysis [47]. CTC are enriched by bead-based approaches [20,48], or the CellSearch^®^ Profile kit and analyzed by subsequent qPCR or RNA-seq [26,30]. While these approaches effectively asses *ARV7* status, they give no additional information on the abundance of CTCs in a patient at the time point of blood draw. This could, however, prove to be valuable information allowing more precise interpretation of the qualitative ARV7^+^ or ARV7^−^ status of a patient. Without CTC count, an ARV7^−^ status may refer to no available CTCs within the blood draw or to high amounts of ARV7^−^ CTCs, respectively. The clinical information to be gained from both results is, however, very different, as no CTCs indicate good and ≥5 CTCs indicate poor outcome for the patient [8]. Multiplexing of additional genes such as prostate specific antigen (PSA) or prostate specific membrane antigen (PSMA), as well as epithelial genes is commonly used as a means of circumventing this issue and attempting to detect ARV7^−^ CTCs [20,26,49]. While this is a feasible approach, it is limited by heterogeneous expression of these markers [31,41,49,50,51] and the required pre-amplification step can introduce bias.

Using our novel approach (Figure 4) information on both CTC count, AR-FL and *ARV7* status is collected. One could argue that the amount of CTCs present in the blood tube destined for *ARV7* assessment is also not directly assessed by our assay. However, studies have shown that CTC counts determined with the CellSearch^®^ technology do not significantly fluctuate depending on circadian rhythm or serial blood draws [52,53], thereby indicating that stochastically, similar to equal CTC amounts would be expected in two sequential blood draws from the same patient at the same time (as is necessitated by our protocol). The importance of integrated CTC enumeration becomes apparent when looking at clinical cases such as UKE-23 (Table 4). While this patient had clearly detectable *ARV7* transcripts, he did not show *K19* positivity in our assay. Without the additional information of 22 CTCs being detected via CellSearch^®^ analysis, interpretation of the qPCR results would have been impaired. This case also highlights the inert limitation of qPCR multiplexing, which lies in the before mentioned heterogeneity of gene and protein expression in CTCs [41,50,51]. In addition, CTC detection via the CellSearch^®^ allows for morphological assessment of the CTCs in circulation and in our case, parallel characterization of AR-FL protein as well as its cellular location. Both represent important factors in resistance to androgen deprivation therapy [54]. The localization of the full length AR within the cell has been shown to be associated with disease progression on novel hormone therapies (e.g., enzalutamide and abiraterone) [55]. Therefore, it was critical for our assay to be able to distinguish both cytoplasmic and nuclear fractions of AR to support differentiation between “AR-on” and “AR-off” patients [54]. Apart from the AR-FL targeting antibody (by Janssen Diagnostics) used in this study, other well-established alternative antibodies have been published for AR-FL detection in the fourth channel of the CellSearch^®^ [55].

To our knowledge, only two assays have been developed allowing parallel CTC enumeration and ARV7 protein [24,25] or transcript detection [31] on the same cell so far. El-Heliebi et al. isolated CTCs via the CellSearch^®^ Profile kit or the size-dependent Parsortix™ platform (ANGLE plc, Guildford, UK) [56] and subsequently characterized them for *ARV7, AR-FL*, and *PSA* expression via in situ padlock probe technology [31]. This approach allows for absolute transcript quantification while keeping cell morphology intact and thereby enabling tumor cell enumeration [31]. In regards to CellSearch^®^ Profile kit pre-enrichment, a single patient with high CTC load was included in this study to demonstrate general feasibility of the approach [31]. Additional technical validation will therefore be required to ensure sufficient sensitivity and specificity of this method for future clinical application.

The ARV7 assay most advanced in regards to clinical validation is the EPICs approach [24,25]. Here, the nuclear cell fraction is placed on slides, stained via ICC and automatically screened and evaluated. The assay focusses on nuclear ARV7 protein expression using the same antibody clone EPR15656 (Abcam) that we tested in our present study. While the EPICS approach allows for parallel CTC enumeration and ARV7 protein assessment, it requires sample shipment to a centralized lab in the US, a costs intensive approach when conducting larger clinical studies or when shipping patient samples for routine testing. A nuclear ARV7 staining has been postulated to be relevant to predict therapy outcome of AR-targeted therapies as well as taxanes in a cohort of 161 mCRPC patients, leading to a favorable coverage recommendation and certification of the approach in the state of California (USA) [57,58]. However, in our hands, the EPR15656 antibody did not result in specific nuclear staining signals for tested cancer cell line cells on chamber slides or cytospins (Figure 1, Figure S1).

While the *ARV7* detection assay established in this study is highly specific and sensitive, some limitations require mentioning. The main limitation is the fact that our assay does not allow for simultaneous morphological and molecular *ARV7* characterization of each single CTC. However, this is somewhat compensated by the use of a clinically validated CTC enrichment method, adding weight to the clinical relevance of the CTCs analyzed. Additionally, *ARV7* and *K19* transcript detection cannot be guaranteed down to a single CTC level in all patient samples. Nevertheless, we can secure determination of *ARV7* status from ≥5 CTCs which is the prognostic cut-off value for patients with metastatic prostate cancer. Due to the high sensitivity and specificity of our *ARV7* detection assay and the parallel nature of the CellSearch^®^ CTC-enumeration, *K19* detection is not a mandatory prerequisite for robust *ARV7* assessment and result interpretation. However, we believe *K19* adds further valuable information in positive cases and represents an additional confirmation of successful CTC analysis.

Taken together, the use of a FDA-cleared enrichment technology, high assay sensitivity and specificity, a shipment window of 24 h and comparably low necessity of elaborate additional laboratory equipment (standard qPCR cycler) corroborate the value of our established method. Inclusion into prospective clinical trials will be now necessary to demonstrate clinical validity and utility. Furthermore, additional age-matched healthy donors and other control cohorts (e.g., prostatitis patients) should be included into future studies to further corroborate assay specificity. Head-to-head comparison with other ARV7/CTC technologies is desirable to assess to which extent different assays are redundant or complementary.

## 5. Conclusions

The novel workflow developed in this study allows for a semi-automated enrichment of CTCs followed by a qPCR assay measuring the *ARV7* status of CTCs. This approach can now be integrated into future clinical trials assessing treatment responses to antiandrogen therapies in prostate cancer patients.

## Figures and Tables

**Table 1:** Validation of protocol specificity and sensitivity. Titration experiments of spiked cell lines in blood from healthy donor (HD) samples. Indicated cell counts of *ARV7*^+^ (22Rv1) and *ARV7*^−^ (PC3) cells were manually spiked into HD blood and processed by our protocol. *ARV7* status is highlighted as “+” for positive and “−“ for negative samples. Detection of gene expression was confirmed when at least 2/3 triplicates were positive in qPCR analysis. Unamplified qPCR samples are marked as N/D (not detected). HD samples were processed in parallel as a negative control for *ARV7* and *K19*. The bar (-) in the respective table column of detection indicates no further experiments were conducted.

Cell Line	*ARV7* Status	Cell Amount	Target	Detection	Detection (n = 2)	Detection (n = 3)
HD	−	0	*ARV7*	N/D	N/D	N/D
			*K19*	N/D	N/D	N/D
			*Actin*	yes	yes	yes
PC3	−	50	*ARV7*	N/D	-	-
			*K19*	yes	-	-
			*Actin*	yes	-	-
		100	*ARV7*	N/D	-	-
			*K19*	yes	-	-
			*Actin*	yes	-	-
22RV1	+	50	*ARV7*	yes	-	-
			*K19*	yes	-	-
			*Actin*	yes	-	-
		20	*ARV7*	yes	-	-
			*K19*	yes	-	-
			*Actin*	yes	-	-
		10	*ARV7*	yes	yes	yes
			*K19*	yes	yes	yes
			*Actin*	yes	yes	yes
		5	*ARV7*	yes	yes	yes
			*K19*	yes	yes	yes
			*Actin*	yes	yes	yes

**Table 2:** Validation of protocol specificity and sensitivity after 24 h. Influence of sample storage on *ARV7* detection limits and assay robustness. *ARV7* status is highlighted as “+” for positive and “−“ for negative samples. Detection of gene expression was confirmed when at least 2/3 triplicates were positive in qPCR analysis. N represents the number of repetitions performed per experimental setting. The ratio is defined as the frequency at which any specific gene was detected out of the N repetitions. N/D signifies no gene expression or gene expression above the set threshold of 35 cycles.

Cell Line	ARV7 Status	Cell Amount	Target	Detection	N	Ratio [detection/N]
HD	−	0	*ARV7*	N/D	3	3/3
			*K19*	N/D		
			*Actin*	yes		
22RV1	+	10	*ARV7*	yes	1	1/1
			*K19*	yes		
			*Actin*	yes		
		5	*ARV7*	yes	2	2/2
			*K19*	yes		
			*Actin*	yes		
		3	*ARV7*	yes	4	1/4
			*K19*	yes		3/4
			*Actin*	yes		4/4
		1	*ARV7*	yes	3	1/3
			*K19*	yes		2/3
			*Actin*	yes		3/3
VCaP	+	10	*ARV7*	yes	2	2/2
			*K19*	yes		
			*Actin*	yes		
		5	*ARV7*	yes	1	1/1
			*K19*	N/D		
			*Actin*	yes		

**Table 3:** Influence of sample tubes and sample storage times on ARV7 detection limits and assay specificity. *ARV7* status is highlighted as “+” for positive and “−“ for negative samples. Detection of gene expression was confirmed when at least 2/3 triplicates were positive in qPCR analysis. N represents the number of repetitions performed per experimental setting. The ratio is defined as the frequency at which any specific gene was detected out of the N repetitions. N/D signifies no gene expression or gene expression above the set threshold of 35 cycles.

Tube	Cell Line	ARV7 Status	Cell Amount	Target	Detection	N	Ratio [detection/N]
EDTA	HD	−	0	*ARV7*	N/D	3	3/3
				*K19*	N/D		
				*Actin*	yes		
	22RV1	+	10	*ARV7*	N/D	1	1/1
				*K19*	yes		
				*Actin*	yes		
			5	*ARV7*	yes	3	2/3
				*K19*	yes		2/3
				*Actin*	yes		3/3
Adnagen	HD	−	0	*ARV7*	yes	3	1/3
				*K19*	yes		2/3
				*Actin*	yes		3/3
	22RV1	+	10	*ARV7*	N/D	1	1/1
				*K19*	N/D		
				*Actin*	yes		
			5	*ARV7*	yes	3	3/3
				*K19*	yes		1/3
				*Actin*	yes		3/3

**Table 4:** Correlation of qPCR results, AR-FL detection and CTC enumeration via CellSearch for 26 mPCa patients analyzed. Detection of a gene was confirmed when at least 2/3 triplicates were positive in qPCR analysis. N/D signifies no gene expression or gene expression above the set threshold of 35 cycles. CTC enumeration via CellSearch^®^ was not conducted for the first three patient samples, indicated by a bar in the respective table column (-). This also applies to 14 samples collected regarding AR occurrence. The number of CTCs with detectable AR-FL expression is indicated in brackets.

		CellSearch		qPCR		
Sample	CTC Count	AR (nucl.)	AR (cytopl.)	*ARV7*	*K19*	*Actin*
UKE-1	-	-	-	yes	yes	yes
UKE-2	-	-	-	N/D	N/D	yes
UKE-3	-	-	-	N/D	N/D	yes
UKE-4	0	-	-	N/D	N/D	yes
UKE-5	0	-	-	N/D	N/D	yes
UKE-6	0	-	-	N/D	N/D	yes
UKE-7	0	0	0	N/D	N/D	yes
UKE-8	1	0	yes (1)	N/D	N/D	yes
UKE-9	1	yes (1)	0	N/D	N/D	yes
UKE-10	1	0	0	N/D	yes	yes
UKE-11	1	0	yes (1)	N/D	yes	yes
UKE-12	1	-	-	yes	yes	yes
UKE-13	1	0	0	yes	yes	yes
UKE-14	2	yes (1)	yes (1)	N/D	yes	yes
UKE-15	6	0	0	N/D	N/D	yes
UKE-16	6	-	-	N/D	N/D	yes
UKE-17	8	yes (3)	yes (4)	yes	yes	yes
UKE-18	9	0	yes (9)	yes	yes	yes
UKE-19	11	0	yes (11)	yes	yes	yes
UKE-20	11	0	yes (11)	yes	yes	yes
UKE-21	14	-	-	N/D	yes	yes
UKE-22	16	-	-	yes	yes	yes
UKE-23	22	-	-	yes	N/D	yes
UKE-24	80	-	-	yes	yes	yes
UKE-25	156	-	-	yes	yes	yes
UKE-26	398	-	-	yes	yes	yes

## Supplementary Materials

The following are available online at https://www.mdpi.com/2073-4409/8/9/1067/s1. Figure S1: Assessment of ARV7 antibody performance on selected prostate cancer cell lines via Western Blot and immunocytochemical (ICC) staining; Table S1: Clinical data of 26 mPCa patients.

## References

[1] World Cancer Research Fund Inthernational, American Institute for Cancer Research (2018). Prostate Cancer. https://www.wcrf.org/dietandcancer/prostate-cancer.

[2] Ladjevardi S., Auer G., Castro J., Ericsson C., Zetterberg A., Häggman M., Wiksell H., Jorulf H. (2014). Prostate Biopsy Sampling Causes Hematogenous Dissemination of Epithelial Cellular Material. Dis. Markers.

[3] Alix-Panabières C., Pantel K. (2016). Clinical Applications of Circulating Tumor Cells and Circulating Tumor DNA as Liquid Biopsy. Cancer Discov..

[4] Pantel K., Speicher M.R. (2016). The biology of circulating tumor cells. Oncogene.

[5] Bardelli A., Pantel K. (2017). Liquid Biopsies, What We Do Not Know (Yet). Cancer Cell.

[6] Alix-Panabieres C., Pantel K. (2013). Circulating tumor cells: Liquid biopsy of cancer. Clin. Chem..

[7] Hille C., Pantel K. (2018). Circulating tumour cells in prostate cancer. Nat. Rev. Urol..

[8] De Bono J.S., Scher H.I., Montgomery R.B., Parker C., Miller M.C., Tissing H., Doyle G.V., Terstappen L.W.W.M., Pienta K.J., Raghavan D. (2008). Circulating tumor cells predict survival benefit from treatment in metastatic castration-resistant prostate cancer. Clin. Cancer Res..

[9] Scher H.I., Jia X.Y., de Bono J.S., Fleisher M., Pienta K.J., Raghavan D., Heller G. (2009). Circulating tumour cells as prognostic markers in progressive, castration-resistant prostate cancer: A reanalysis of IMMC38 trial data. Lancet. Oncol..

[10] Scher H.I., Heller G., Molina A., Attard G., Danila D.C., Jia X., Peng W., Sandhu S.K., Olmos D., Riisnaes R. (2015). Circulating Tumor Cell Biomarker Panel As an Individual-Level Surrogate for Survival in Metastatic Castration-Resistant Prostate Cancer. J. Clin. Oncol..

[11] Heller G., Fizazi K., McCormack R., Molina A., MacLean D., Webb I.J., Saad F., de Bono J.S., Scher H.I. (2017). The Added Value of Circulating Tumor Cell Enumeration to Standard Markers in Assessing Prognosis in a Metastatic Castration-Resistant Prostate Cancer Population. Clin. Cancer Res..

[12] Miller M.C., Doyle G.V., Terstappen L.W. (2010). Significance of Circulating Tumor Cells Detected by the CellSearch System in Patients with Metastatic Breast Colorectal and Prostate Cancer. J. Oncol..

[13] Goodman O.B., Symanowski J.T., Loudyi A., Fink L.M., Ward D.C., Vogelzang N.J. (2011). Circulating Tumor Cells as a Predictive Biomarker in Patients With Hormone-sensitive Prostate Cancer. Clin. Genitourin. Cancer.

[14] Danila D.C., Fleisher M., Scher H.I. (2011). Circulating tumor cells as biomarkers in prostate cancer. Clin. Cancer Res..

[15] Gorges T.M., Pantel K. (2013). Circulating tumor cells as therapy-related biomarkers in cancer patients. Cancer Immunol. Immunother..

[16] Singhal U., Wang Y., Henderson J., Niknafs Y.S., Qiao Y., Gursky A., Zaslavsky A., Chung J.-S., Smith D.C., Karnes R.J. (2018). Multigene Profiling of CTCs in mCRPC Identifies a Clinically Relevant Prognostic Signature. Mol. Cancer Res..

[17] Pantel K., Hille C., Scher H.I. (2019). Circulating Tumor Cells in Prostate Cancer: From Discovery to Clinical Utility. Clin. Chem..

[18] Luo J., Attard G., Balk S.P., Bevan C., Burnstein K., Cato L., Cherkasov A., De Bono J.S., Dong Y., Gao A.C. (2018). Role of Androgen Receptor Variants in Prostate Cancer: Report from the 2017 Mission Androgen Receptor Variants Meeting. Eur. Urol..

[19] Antonarakis E.S., Armstrong A.J., Dehm S.M., Luo J. (2016). Androgen receptor variant-driven prostate cancer: Clinical implications and therapeutic targeting. Prostate Cancer Prostatic Dis..

[20] Antonarakis E.S., Lu C., Wang H., Luber B., Nakazawa M., Roeser J.C., Chen Y., Mohammad T.A., Chen Y., Fedor H.L. (2014). AR-V7 and Resistance to Enzalutamide and Abiraterone in Prostate Cancer. New Engl. J. Med..

[21] Antonarakis E.S., Luo J. (2016). Blood Based Detection of Androgen Receptor Splice Variants in Patients with Advanced Prostate Cancer. J. Urol..

[22] Lokhandwala P.M., Riel S.L., Haley L., Lu C., Chen Y., Silberstein J., Zhu Y., Zheng G., Lin M.-T., Gocke C.D. (2017). Analytical Validation of Androgen Receptor Splice Variant 7 Detection in a Clinical Laboratory Improvement Amendments (CLIA) Laboratory Setting. J. Mol. Diagn..

[23] Antonarakis E.S., Lu C., Luber B., Wang H., Chen Y., Zhu Y., Silberstein J.L., Taylor M.N., Maughan B.L., Denmeade S.R. (2017). Clinical Significance of Androgen Receptor Splice Variant-7 mRNA Detection in Circulating Tumor Cells of Men With Metastatic Castration-Resistant Prostate Cancer Treated With First- and Second-Line Abiraterone and Enzalutamide. J. Clin. Oncol..

[24] Scher H.I., Graf R.P., Schreiber N.A., Jayaram A., Winquist E., McLaughlin B., Lu D., Fleisher M., Orr S., Lowes L. (2018). Assessment of the Validity of Nuclear-Localized Androgen Receptor Splice Variant 7 in Circulating Tumor Cells as a Predictive Biomarker for Castration-Resistant Prostate Cancer. JAMA Oncol..

[25] Scher H.I., Lu D., Schreiber N.A., Louw J., Graf R.P., Vargas H.A., Johnson A., Jendrisak A., Bambury R., Danila D. (2016). Association of AR-V7 on Circulating Tumor Cells as a Treatment-Specific Biomarker With Outcomes and Survival in Castration-Resistant Prostate Cancer. JAMA Oncol..

[26] Onstenk W., Sieuwerts A.M., Kraan J., Van M., Nieuweboer A.J., Mathijssen R.H., Hamberg P., Meulenbeld H.J., De Laere B., Dirix L.Y. (2015). Efficacy of Cabazitaxel in Castration-resistant Prostate Cancer Is Independent of the Presence of AR-V7 in Circulating Tumor Cells. Eur. Urol..

[27] Antonarakis E.S., Lu C., Luber B., Wang H., Chen Y., Nakazawa M., Nadal R., Paller C.J., Denmeade S.R., Carducci M.A. (2015). Androgen Receptor Splice Variant 7 and Efficacy of Taxane Chemotherapy in Patients With Metastatic Castration-Resistant Prostate Cancer. JAMA Oncol..

[28] Bernemann C., Schnoeller T.J., Luedeke M., Steinestel K., Boegemann M., Schrader A.J., Steinestel J. (2017). Expression of AR-V7 in Circulating Tumour Cells Does Not Preclude Response to Next Generation Androgen Deprivation Therapy in Patients with Castration Resistant Prostate Cancer. Eur. Urol..

[29] Nakazawa M., Lu C., Chen Y., Paller C.J., Carducci M.A., Eisenberger M.A., Luo J., Antonarakis E.S. (2015). Serial blood-based analysis of AR-V7 in men with advanced prostate cancer. Ann. Oncol..

[30] De Laere B., Van Dam P.-J., Whitington T., Mayrhofer M., Diaz E.H., Eynden G.V.D., Vandebroek J., Del-Favero J., Van Laere S., Dirix L. (2017). Comprehensive Profiling of the Androgen Receptor in Liquid Biopsies from Castration-resistant Prostate Cancer Reveals Novel Intra-AR Structural Variation and Splice Variant Expression Patterns. Eur. Urol..

[31] El-Heliebi A., Attard G., Balk S.P., Bevan C., Burnstein K., Cato L., Cherkasov A., De Bono J.S., Dong Y., Gao A.C. (2018). In Situ Detection and Quantification of AR-V7, AR-FL, PSA, and KRAS Point Mutations in Circulating Tumor Cells. Clin. Chem..

[32] Sharp A., Welti J.C., Lambros M.B., Dolling D., Rodrigues D.N., Pope L., Aversa C., Figueiredo I., Fraser J., Ahmad Z. (2019). Clinical Utility of Circulating Tumour Cell Androgen Receptor Splice Variant-7 Status in Metastatic Castration-resistant Prostate Cancer. Eur. Urol..

[33] Riethdorf S., O’Flaherty L., Hille C., Pantel K. (2018). Clinical applications of the CellSearch platform in cancer patients. Adv. Drug Deliv. Rev..

[34] Riethdorf S., Fritsche H., Müller V., Rau T., Schindlbeck C., Rack B., Janni W., Coith C., Beck K., Jänicke F. (2007). Detection of Circulating Tumor Cells in Peripheral Blood of Patients with Metastatic Breast Cancer: A Validation Study of the CellSearch System. Clin. Cancer Res..

[35] Guo Z., Yang X., Sun F., Jiang R., Linn D.E., Chen H., Chen H., Kong X., Melamed J., Tepper C.G. (2009). A novel androgen receptor splice variant is up-regulated during prostate cancer progression and promotes androgen depletion-resistant growth. Cancer Res..

[36] Liu L.L., Xie N., Sun S., Plymate S., Mostaghel E., Dong X. (2014). Mechanisms of the androgen receptor splicing in prostate cancer cells. Oncogene.

[37] Untergasser A., Cutcutache I., Koressaar T., Ye J., Faircloth B.C., Remm M., Rozen S.G. (2012). Primer3—New capabilities and interfaces. Nucleic Acids Res..

[38] Livak K.J., Schmittgen T.D. (2001). Analysis of relative gene expression data using real-time quantitative PCR and the 2(-Delta Delta C(T)) Method. Methods.

[39] Stathopoulou A., Angelopoulou K., Perraki M., Georgoulias V., Malamos N., Lianidou E. (2001). Quantitative RT-PCR luminometric hybridization assay with an RNA internal standard for cytokeratin-19 mRNA in peripheral blood of patients with breast cancer. Clin. Biochem..

[40] Stathopoulou A., Gizi A., Perraki M., Apostolaki S., Malamos N., Mavroudis D., Georgoulias V., Lianidou E.S. (2003). Real-time quantification of CK-19 mRNA-positive cells in peripheral blood of breast cancer patients using the lightcycler system. Clin. Cancer Res..

[41] Gorges T.M., Kuske A., Röck K., Mauermann O., Müller V., Peine S., Verpoort K., Novosadova V., Kubista M., Riethdorf S. (2016). Accession of Tumor Heterogeneity by Multiplex Transcriptome Profiling of Single Circulating Tumor Cells. Clin. Chem..

[42] Cristofanilli M., Budd G.T., Ellis M.J., Stopeck A., Matera J., Miller M.C., Reuben J.M., Doyle G.V., Allard W.J., Terstappen L.W. (2004). Circulating Tumor Cells, Disease Progression, and Survival in Metastatic Breast Cancer. New Engl. J. Med..

[43] Cohen S.J., Punt C.J., Iannotti N., Saidman B.H., Sabbath K.D., Gabrail N.Y., Picus J., Morse M., Mitchell E., Miller M.C. (2008). Relationship of Circulating Tumor Cells to Tumor Response, Progression-Free Survival, and Overall Survival in Patients With Metastatic Colorectal Cancer. J. Clin. Oncol..

[44] Ligthart S.T., Coumans F.A.W., Attard G., Cassidy A.M., De Bono J.S., Terstappen L.W.M.M. (2011). Unbiased and Automated Identification of a Circulating Tumour Cell Definition That Associates with Overall Survival. PLoS ONE.

[45] Scholtens T.M., Schreuder F., Ligthart S.T., Swennenhuis J.F., Greve J., Terstappen L.W. (2012). Automated identification of circulating tumor cells by image cytometry. Cytometry A.

[46] Markowski M.C., Silberstein J.L., Eshleman J.R., Eisenberger M.A., Luo J., Antonarakis E.S. (2017). Clinical Utility of CLIA-Grade AR-V7 Testing in Patients With Metastatic Castration-Resistant Prostate Cancer. JCO Precis. Oncol..

[47] Todenhöfer T., Azad A., Stewart C., Gao J., Eigl B.J., Gleave M.E., Joshua A.M., Black P.C., Chi K.N. (2017). AR-V7 Transcripts in Whole Blood RNA of Patients with Metastatic Castration Resistant Prostate Cancer Correlate with Response to Abiraterone Acetate. J. Urol..

[48] Tommasi S., Pilato B., Carella C., Lasorella A., Danza K., Vallini I., De Summa S., Naglieri E. (2018). Standardization of CTC AR-V7 PCR assay and evaluation of its role in castration resistant prostate cancer progression. Prostate.

[49] Sieuwerts A.M., Mostert B., Van Der Vlugt-Daane M., Kraan J., Beaufort C.M., Van M., Prager W.J., De Laere B., Beije N., Hamberg P. (2018). An In-Depth Evaluation of the Validity and Logistics Surrounding the Testing of AR-V7 mRNA Expression in Circulating Tumor Cells. J. Mol. Diagn..

[50] Gorges T.M., Riethdorf S., Von Ahsen O., Nastały P., Röck K., Boede M., Peine S., Kuske A., Schmid E., Kneip C. (2016). Heterogeneous PSMA expression on circulating tumor cells—A potential basis for stratification and monitoring of PSMA-directed therapies in prostate cancer. Oncotarget.

[51] Scher H.I., Graf R.P., Schreiber N.A., McLaughlin B., Jendrisak A., Wang Y., Lee J., Greene S., Krupa R., Lu D. (2017). Phenotypic Heterogeneity of Circulating Tumor Cells Informs Clinical Decisions between AR Signaling Inhibitors and Taxanes in Metastatic Prostate Cancer. Cancer Res..

[52] Martín M., García-Sáenz J.A., Casas M.L.M.D.L., Vidaurreta M., Puente J., Veganzones S., Rodríguez-Lajusticia L., De La Orden V., Oliva B., De La Torre J.-C. (2009). Circulating tumor cells in metastatic breast cancer: timing of blood extraction for analysis. Anticancer. Res..

[53] Tibbe A.G.J., Miller M.C., Terstappen L.W.M.M. (2007). Statistical considerations for enumeration of circulating tumor cells. Cytom. Part. A.

[54] Miyamoto D.T., Lee R.J., Stott S.L., Ting D.T., Wittner B.S., Ulman M., Smas M.E., Lord J.B., Brannigan B.W., Trautwein J. (2012). Androgen receptor signaling in circulating tumor cells as a marker of hormonally responsive prostate cancer. Cancer Discov..

[55] Crespo M., Van Dalum G., Ferraldeschi R., Zafeiriou Z., Sideris S., Lorente D., Bianchini D., Rodrigues D.N., Riisnaes R., Miranda S. (2015). Androgen receptor expression in circulating tumour cells from castration-resistant prostate cancer patients treated with novel endocrine agents. Br. J. Cancer.

[56] Hvichia G., Parveen Z., Wagner C., Janning M., Quidde J., Stein A., Müller V., Loges S., Neves R., Stoecklein N. (2016). A novel microfluidic platform for size and deformability based separation and the subsequent molecular characterization of viable circulating tumor cells. Int. J. Cancer.

[57] (2018). Services, C.f.M.M.. https://med.noridianmedicare.com/documents/10546/6990981/MolDX+Circulating+Tumor+Cell+Marker+Assays+LCD/8eaf89f0-9970-455c-b048-ebaeaf42bd7d..

[58] (2018). Services C.f.M.M.. https://www.cms.gov/medicare-coverage-database/details/lcd-details.aspx?LCDId=37914&ver=2&Cntrctr=All&UpdatePeriod=796&bc=AQAAEAAAAAAA&..

