# Supplementary material for: Detection of Androgen Receptor Variant 7 (ARV7) mRNA Levels in EpCAM-Enriched CTC Fractions for Monitoring Response to Androgen Targeting Therapies in Prostate Cancer
Source: Cells. 2019 Sep 11;8(9):1067. doi: 10.3390/cells8091067 (PMC6770695; doi:10.3390/cells8091067)
Supplement: Supplementary file 1 [file cells-08-01067-s001.pdf]

Supplementary Figure S1:

(a)

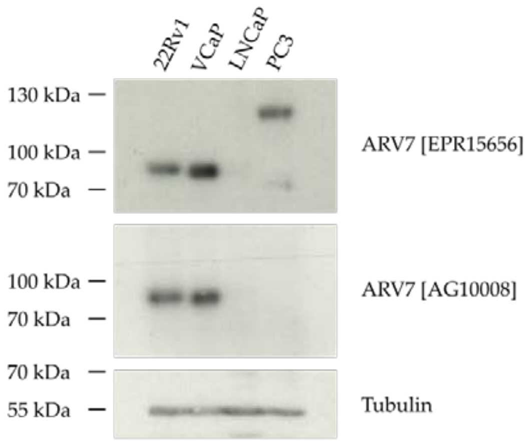

(b)

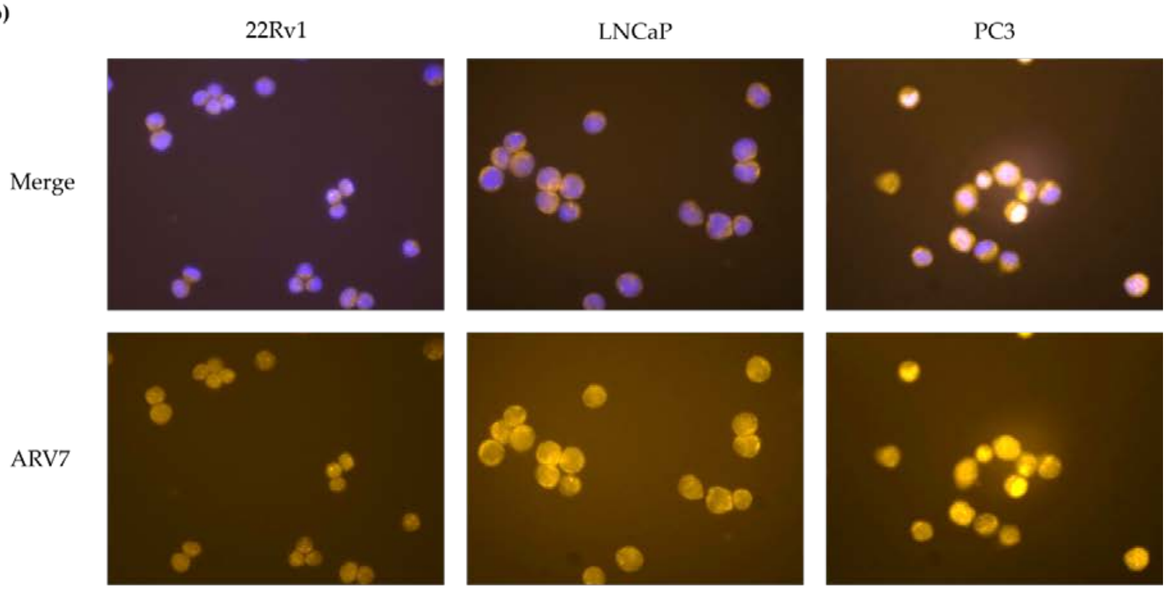

(c)

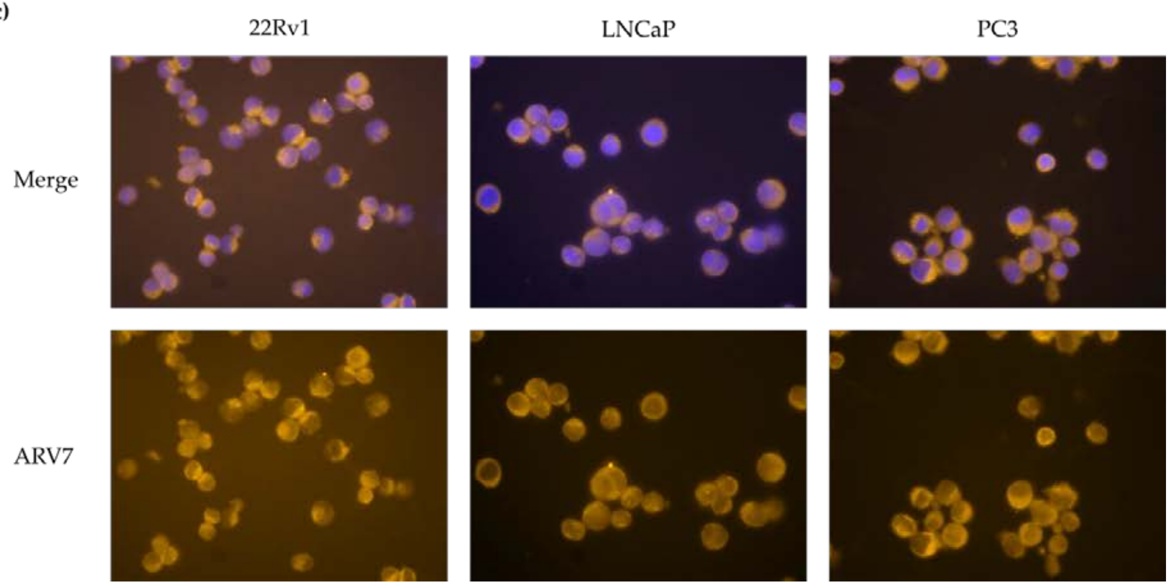

Figure S1: Assessment of ARV7 antibody performance on selected prostate cancer cell lines via Western Blot and immunocytochemical (ICC) staining. **(a)** Western Blot analysis of antibodies EPR15656 and AG10008. 40µg of total protein from four prostate cancer cell lines were applied. Tubulin was used as a loading control. **(b)** ICC of ARV7 on three selected prostate cancer cell lines. Upper panel: merged images, lower panel: ARV7 staining. Cells on cytospins are stained using the EPR15656 clone targeting ARV7 with a species specific secondary antibody in red (Alexa 546).. The nucleus is counterstained with DAPI. Pictures are taken at 40x magnification. **(c)** ICC of ARV7 on three selected prostate cancer cell lines. Upper panel: merged images, lower panel: ARV7 staining. Cells on cytospins are stained using the AG10008 clone targeting ARV7 with a species specific secondary antibody in red (Alexa 546). The nucleus is counterstained with DAPI. Pictures are taken at 40x magnification. Both **(b)** and **(c)** show unspecific staining results for each ARV7-targeting antibody respectively. Especially for clone EPR15656 **(b)** nuclear staining in ARV7^{-} PC3 cells appears to be more intense than for ARV7^{+} 22Rv1 cells.

Table S1: Clinical data of 26 mPCa patients. Non-available data indicated as “n.a.”.

1 = yes

0 = no

**Patient Cohort**																											
**Patient Characteristics**	**All Patients**	**UKE-1**	**UKE-2**	**UKE-3**	**UKE-4**	**UKE-5**	**UKE-6**	**UKE-7**	**UKE-8**	**UKE-9**	**UKE-10**	**UKE-11**	**UKE-12**	**UKE-13**	**UKE-14**	**UKE-15**	**UKE-16**	**UKE-17**	**UKE-18**	**UKE-19**	**UKE-20**	**UKE-21**	**UKE-22**	**UKE-23**	**UKE-24**	**UKE-25**	**UKE-26**
**Unique number of patients**	26	1	1	1	1	1	1	1	1	1	1	1	1	1	1	1	1	1	1	1	1	1	1	1	1	1	1
**Age upon blood draw [years]**	68	76	78	65	77	58	71	59	70	78	70	67	73	60	73	74	42	59	73	74	66	65	69	76	65	64	70
**Primary treatment, Number (%)**																											
Prostatectomy (surgery)	10	0	0	0	0	1	0	1	1	1	1	1	0	0	0	1	0	0	0	1	1	1	0	0	0	1	0
Radiation	6	1	0	0	1	0	0	0	1	0	0	0	1	0	1	0	0	1	0	1	0	0	0	0	0	0	1
None	3	0	0	1	0	0	0	0	0	0	0	0	0	0	0	0	1	0	1	0	0	0	1	0	0	0	0
**Tumor stage at diagnosis, Number (%)**																											
actual number		cT3 pN0 Gl5+5 M1	cT3 NX M1 Gl4+5	n.a.	cT3 N1 Gl 4+5 M0	pT3bp N1 M1 Gl4+5	cT1bGl 4+5 Nx M1	pT3b pN1 R1 M0	Pt2c pN1 MoRo L1V0	pT2c, N0, M0	n.a.	PT3a, pN0	cT3 M1 NX Gl 4+5	M1	T2a	pT3a N0 R0 29	ypT1b NX M1	T4	n.a.	PT3b, pN1, R1	PT3b, pN1	pT4 pN1M 1Gl 4+5	n.a.	cT3N M1 Gl. 4+5	cT3 NX M1 Gl5+4	pT3a R1 pN1	T1c N0 M0

T1/T2	5	0	0	0	0	0	1	0	1	1	0	0	0	0	1	0	1	0	0	0	0	0	0	0	0	0	1
T3/T4	11	1	1	0	1	1	0	1	0	0	0	1	1	0	0	1	0	1	0	1	1	1	0	1	1	1	0
**Gleason sum at diagnosis, Number (%)**																											
≤7	7	0	0	0	0	0	0	0	1	1	1	0	0	0	1	0	1	0	1	0	0	0	0	0	0	0	1
≥8	12	1	1	1	1	1	1	1	0	0	0	1	1	1	0	0	0	1	0	1	1	1	1	1	1	1	0
unknown	1	0	0	0	0	0	0	0	0	0	0	0	0	0	0	1	0	0	0	0	0	0	0	0	0	0	0
**Samples Characteristics**																											
**Prior to blood collection, use of abiraterone**																											
Yes	5	0	0	0	0	0	0	0	1	0	1	0	0	0	0	0	0	0	1	0	0	0	0	0	1	1	0
No	15	1	1	1	1	1	1	1	0	1	0	1	1	1	1	1	1	1	0	1	1	1	1	1	0	0	1
**Prior to blood collection, use of enzalutamide**																											
Yes	5	1	0	0	0	0	0	0	0	0	0	0	0	0	0	0	0	0	1	0	0	0	1	0	1	1	1
No	15	0	1	1	1	1	1	1	1	1	1	1	1	1	1	1	1	1	0	1	1	1	0	1	0	0	0
**Prior to blood collection, use of Docetaxel Chemotherapy**																											
Yes	10	1	0	0	0	0	1	0	1	0	1	0	0	1	0	0	1	0	1	0	0	0	1	1	1	1	1
No	10	0	1	1	1	1	0	1	0	1	0	1	1	0	1	1	0	1	0	1	1	1	0	0	0	0	0
**Prior to blood**																											

**collection, use of Cabazitaxel Chemotherapy**																											
Yes	3	0	0	0	0	0	0	0	0	0	0	0	0	0	0	0	0	0	0	0	0	0	1	0	0	1	1
No	17	1	1	1	1	1	1	1	1	1	1	1	1	1	1	1	1	1	1	1	1	1	0	1	1	0	0
**Threatment line before blood collection, Number (%)**																											
First line	12	0	1	1	0	0	0	1	1	1	1	1	0	1	1	1	1	1	0	1	1	0	0	0	0	0	0
Second line	3	0	0	0	1	1	1	0	0	0	0	0	1	0	0	0	0	0	0	0	0	1	0	1	0	0	0
Third line or later	5	1	0	0	0	0	0	0	0	0	0	0	0	0	0	0	0	0	1	0	0	0	1	0	1	1	1
**Metastatic disease, Number (%)**																											
Bone	20	1	1	1	0	1	1	1	1	1	1	1	1	1	1	1	1	1	1	1	1	1	1	1	1	1	1
liver	1	0	0	0	0	0	0	0	0	0	0	0	0	0	0	0	0	0	0	0	0	0	1	0	0	0	0
Lymph node	10	1	0	0	1	1	0	0	1	1	0	0	0	0	1	0	1	1	0	0	1	1	1	0	0	1	1
Other	2	1	0	0	0	0	0	0	0	0	0	0	0	0	0	0	0	0	1	0	0	0	0	0	0	1	0
None	0	0	0	0	0	0	0	0	0	0	0	0	0	0	0	0	0	0	0	0	0	0	0	0	0	0	0
unknown	0	0	0	0	0	0	0	0	0	0	0	0	0	0	0	0	0	0	0	0	0	0	0	0	0	0	0
**Laboratory measures, median (range)**	0																										
PSA (at time of blood draw)	2153.72	1106	0.02	3.02	12.27	31.24	50.83	9.51	74.1	10.8	75	2.87	0	68.3	30.1	52.23	1.9	13	571	5.4	16.3	11.5	7.81	54.77	86.13	111	952

**Present status (July 2019)**																											
Alive	4	0	0	1	1	0	1	1	0	0	0	0	0	1	0	0	1	0	0	0	0	0	0	1	0	0	0
Deceased	9	1	1	0	0	1	0	0	0	1	0	0	1	0	0	0	0	0	1	1	0	1	1	0	1	1	1
Unknown	7	0	0	0	0	0	0	0	1	0	1	1	0	0	1	1	0	1	0	0	1	0	0	0	0	0	0
